# 3D Printing Soft Magnet: Binder Study for Vat Photopolymerization of Ferrosilicon Magnetic Composites

**DOI:** 10.3390/polym15163482

**Published:** 2023-08-20

**Authors:** Leah Okoruwa, Farzaneh Sameni, Pavel Borisov, Ehsan Sabet

**Affiliations:** 1 Wolfson School of Mechanical, Electrical and Manufacturing Engineering, Loughborough University, Loughborough LE11 3TU, UK; l.okoruwa@lboro.ac.uk; 2 Additive Manufacturing Centre of Excellence Ltd., Derby DE23 8YH, UK; f.sameni@am-coe.com; 3 Department of Physics, Loughborough University, Loughborough LE11 3TU, UK; p.borisov@lboro.ac.uk

**Keywords:** vat photopolymerization, soft magnetic composite, ferrosilicon, Fe-6.5 wt%Si alloy, binder development, LCD masking, 3D printing magnets

## Abstract

Liquid Crystal Display (LCD) masking is a 3D printing technique that can produce soft magnetic composite parts to high resolution and complexity for robotics and energy electronics applications. This additive manufacturing technique has the potential to produce larger, lighter-weight, more efficient, and more durable parts for automotive and mechanical applications. This study conducted a binder study to create a low-viscosity and stiff binder capable of loading at least 60 *v*/*v*% Fe-6.5 wt%Si particles. Percolation Theory was applied to anticipate the magnetic interaction of suspended particles. A series of binders were formulated, with adjustments to diluent ratios. The behavior of the binders was assessed by studying their rheological properties, conversion rates, and mechanical properties. A post-cure study was conducted across various energy settings using UV, thermal, and a combination of both energy sources to find the combination that provided the best mechanical properties. As a result, 64 *v*/*v*% Fe-6.5 wt%Si loading was achieved and cured using UV light of 405 nm wavelength. Vibrating Sample Spectroscopy (VSM) was used to characterize the composite’s magnetic behavior, and a significant increase in saturation magnetization and negligible change in coercivity was observed when the added load exceeded the percolation threshold.

## 1. Introduction

Additive manufacturing (AM) ranges across various methods, Binder Jetting; Directed Energy Deposition; Material Extrusion; Material Jetting; Powder Bed Fusion; Sheet Lamination, and, finally, Vat Photopolymerization (VPP) according to ISO/ASTM 52900:2021 [1,2]. VPP is an advanced manufacturing method allowing complex designs to be fabricated [3], which is difficult and, in some cases, not easily feasible to the same degree of intricacy using traditional fabrication methods. VPP creates parts by curing a layer of photocurable material layer by layer of low-layer thicknesses allowing for parts of high resolution to be fabricated with this method [4,5]. 

Additionally, 3D printing has provided the opportunity for composite material fabrication by incorporating solid material into a medium, producing a suspension that can be used to print the desired geometry [6,7]. Adding magnetic particles to a medium introduces 4D printing, an innovative branch of the already promising 3D printing processes [8,9,10]. Soft magnetic materials such as electrical steel (FeSi) are reactional to their stimulus, external magnetic fields [11]. In the presence of magnetic field strength, electrical steel can change in crystallographic structure (anisotropy) and exhibit other electromagnetic phenomena beneficial to many applications, such as electromotors, transformers, generators, and robotics [12]. 

Currently, there are limitations to the geometries achievable when producing parts for high-frequency applications using electrical steel. The grade of electrical steel commonly used is Fe-3 wt%Si, a grade of FeSi with high magnetization strength. However, this grade exhibits magnetostriction and iron losses, which are not ideal for many applications, as they drop operation efficiency. Using Fe-6.5 wt%Si rids the material of its magnetostrictive behavior and improves the efficiency of parts due to the increased silicon content, providing electrical resistivity [13]. Fe-6.5 wt%Si is difficult to manufacture with the current methods (annealing and cold rolling) as the increased silicon content leads to brittleness [14]. Methods have been incorporated into creating parts with Fe-6.5 wt%Si, such as Chemical Vapor Deposition. However, this method is still considered restrictive, as the silicon diffusion process used to increase the silicon content from 3 wt.% to 6.5 wt.% only impacts the surface layers of the metal, while the core remains at 3 wt.%, creating a magnetostriction gradient which can build internal stresses [15]. 

In the manufacture of magnetic material, the use of direct sintering and melting AM methods poses undesirable changes to the magnetic properties due to their impact on the microstructure of the magnetic particle [16]. With extrusion-based AM methods, the loading material, such as Ferrite, leads to rapid nozzle wearing and clogging if larger nozzle size or wear-resistant materials are not in use [17], limiting particle loading and introducing an increased risk of print failure [18]. However, VPP provides the highest level of dimensional accuracy [16,19] without impacting the ferrite particle microstructure. This AM method also allows for high-complexity geometries of hollowness and varying wall thicknesses, thinner than traditional manufacturing processes. 

VPP uses light in the ultraviolet (UV) range of the electromagnetic spectrum to cure a photocurable combination of monomers (photoresin), resulting in a solidified polymer of desired geometry. VPP can create composite materials where various solid particles can be loaded into a photoresin binder, creating a photocurable suspension (slurry); how the slurry is cured varies depending on the VPP method used. In stereolithography (SLA), a high-intensity laser is used to cure the free layer of binder/suspension [20]. In digital light processing VPP (DLP), the entire layer is cured at once using a projected image of the layer using a digital micromirror (DMD) [21], while LCD VPP masking uses an array of light-emitting diode (LED) UV light source [22,23].

**Figure 1:**
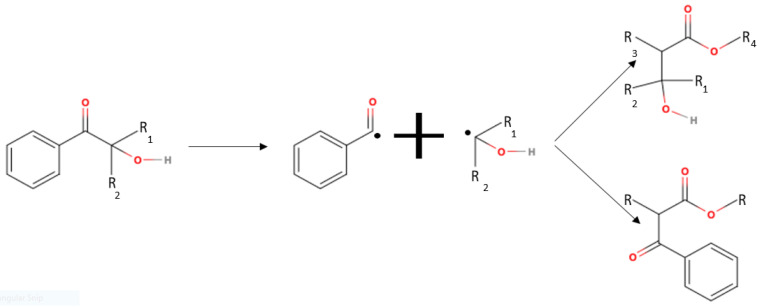
Illustrative reaction mechanism between acrylate monomer and a type I photoinitiator.

A Norrish type I photoinitiator initiates photopolymerization via a homolytic bondage cleavage reaction during polymerization (Figure 1). In this polymerization process, the UV light irradiates and excites the photoinitiator causing the molecule to break into two radicals. The radicals then attack the alkene bond within the acrylate functional group, leading to polymerization. The depletion of the photoinitiator and available bonds for polymerization terminates this mechanism.

The photocurable mixture is offloaded during bottom-up LCD masking into a vat with a clear base. During the printing process, a build platform, suspended above the mixture, is lowered into the vat, stopping with the clearance width equivalent to the specified layer thickness. A UV light source positioned underneath the vat emits UV light of 405 nm [24], causing light-dependent polymerization. The binder cures to a solid state during UV exposure due to crosslinking [25]. Crosslinking is favorable in polymerization when high strength and stiff specimens are desired [26]. Finally, the build platform rises to allow the replenishment of the photocurable mixture at a depth suitable to cure the next layer. At this stage, peeling occurs, where the cured layer detaches from the transparent base film of the vat tray while remaining adhered to the previously printed layer or the build platform [27]. 

The formulation of a binder is designed for suitability for specific applications. A binder typically contains a primary crosslinker, the main component of the formulation, selected based on its properties. These properties manifest in the printed specimen and can be improved by adding other compounds. Primary crosslinkers typically have the highest molecular weight of all components in a binder due to the higher functionality and complexity of the molecule’s structure [28]. This component’s benefit is that it allows covalent and intermolecular bonds to be formed during the polymerization process. The downside to this, however, is the high viscosity, which commonly requires dilution for better printability, especially in particle loading applications [29]. In some high complexity, lower functionality primary crosslinkers, there is a benefit in incorporating a secondary crosslinker, a simple high functionality component to increase the crosslinking density of the formulation. 

High reactivity in binder formulations helps load particles with a higher refractive index as the light scattering effects reduce the total energy utilizable during polymerization, leading to lowered cure depths [30]. Using a more reactive binder maximizes the cure depth attainable in such circumstances.

The loading of highly refractive and light absorptive particles, such as FeSi, causes limitations to the penetration depths of the light source during the curing process. Additionally, with increased particle loading, there is an increase in the viscosity of the slurry. The slurry is no longer printable if the viscosity rises too high (often over 5 Pa·s). Therefore, a suitable binder must be formulated to best suit the intended particle loading and minimize this drawback. 

VPP of magnetic composites have appeared in literature; however, the development of this area of research is at its initial stage. In a recent publication by Hu, Xing et al. [31], a photocurable paste was created with volumetric loading of 49–58% Mn-Zn-Fe for SLA. The paper aimed to print with high particle loading to maximize density and achieve a structure suitable for sintering. Hu explained that with increasing loading of ceramic and magnetic materials, there is an increase in light absorptivity and astigmatism. A design was successfully printed using a 6 wt.% photoinitiator in a low viscosity, tetra functional resin (100–200 mPa·s), and a 355 nm laser was then used for the photopolymerization process. The results of this experiment showed 58% *v*/*v* loading to be the limit, as printing failures could not be avoided past this point. Additionally, with high particle loading, the effects of absorbed light leading to heat generation were magnified to the extent that smoke was visible during printing. However, with successful printing at high loading, the achieved cure depths ranged from 7 to 15 µm, decreasing with increasing load. 

Leigh, Purssell et al. [32] used micro SLA to produce flow sensors using a Magnetite (Fe_3_O_4_)- polymer matrix. This study used micro stereolithography but through an LCD masking technique that created a more rapid printing process. To alleviate aggregation of the magnetite particles (50 nm diameter), a formulation of higher viscosity was created by alternating the ratio of high functionality oligomers to reactive diluents, as opposed to [33,34], who worked with a low viscosity binder and added thixotropic agent to prevent aggregation. A 25 wt.% slurry was created. Loading was limited due to the light-scattering phenomenon occurring with the magnetite loading. The results of this study showed that there was a decrease in mechanical properties when magnetic particle loading was introduced due to disruption in the crosslinking of the monomer.

Nagarajan, Mertiny et al. [35] used DLP to print Strontium Ferrite (SrFeO) and Neodymium magnets (NdFeB), adding two thixotropic agents, Disparlon 6900-20X and BYK-7410 ET, as well as applying sonication to the resin to combat the rate of settlement. However, a low loading of 5 wt.% was used for the study. Therefore, the benefit of using thixotropic agents and sonication was not thoroughly compared to the extent of loading. 

Similar studies are available [33,34], studying VPP-fabricating composites such as ceramics, leaving a gap in the literature concerning knowledge specific to soft magnets, especially FeSi. 

This paper investigated an aspect of photocurable FeSi slurry development to determine the viscosity needed to load at least 60 *v*/*v*% FeSi particles. Currently, the studies related to magnetic slurry development do not explore binder development, detail, or study binder composition’s impact on mechanical properties. Additionally, due to the high density of soft magnetic powder, studies conducted for other composites could not be directly applied to this application. Therefore, a binder study was conducted to find suitable proportions of formulation components to give a low viscous, high stiff mechanically performing binder intended for Fe-6.5 wt%Si particles. The binders were characterized by assessing rheology behavior, cure depth, mechanical properties after curing, and Fourier Transform Infrared-Attenuated Total Reflectance (FTIR-ATR) to assess kinetic behavior.

A post-cure study was performed on the three best-performing binder formulations to enhance the mechanical properties further. In addition to tensile testing, hardness and surface roughness analyses were also conducted. Finally, Fe-6.5 wt%Si loading was performed on the selected binder to achieve a minimum loading of 60 *v*/*v*%. Magnetic testing was conducted on the cured composite using a Vibrating Sample Magnetometer (VSM) to measure a magnetic hysteresis M-H loop. 

## 2. Materials and Methods

### 2.1. Binder Study 

This study used an aliphatic urethane methacrylate oligomer (primary) (Rahn AG, Zürich, Switzerland) as the primary crosslinker. In some formulations, dipentaerythritol hexa acrylate (DPHA) (Rahn AG, Zürich, Switzerland) was used as a secondary crosslinker. However, with the use of aliphatic components instead of aromatic, there is a decrease in thermal stability [36]. Many applications of Fe-6.5 wt%Si are high-frequency and require a frequent directional change of magnetic domains. The result is that heat generation can cause a decrease in efficiency for the magnet and more frequent degradation of the plastic component of the composite. Materials with low thermal conductivity, such as aliphatic plastics, cannot readily dissipate heat, making thermal degradation more likely. However, the magnitude of the heating in question also leads to similar problems in aromatic plastics. Maximizing particle loading can enhance heat dissipation [37] and, therefore, became a prioritized design requirement.

**Figure 2:**
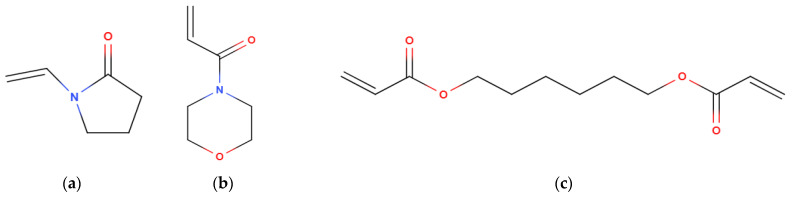
Displayed chemical structure of reactive diluents used in this study. (**a**) N-Vinyl-2-pyrrolidone (NVP). (**b**) 4-Acryloylmorpholine (ACMO) (**c**) 1,6 hexanediol diacrylate (HDDA).

A series of reactive diluents (Table 1) were added to the primary crosslinker to decrease the binder’s viscosity, so more Fe-6.5 wt%Si could be loaded into the system (Figure 2). Each diluent used had varying structures that bring attributes from which the polymer system can benefit. 1,6 hexanediol diacrylate (HDDA) (Rahn AG, Zürich, Switzerland) is an aliphatic difunctional reactive diluent used in formulations to decrease viscosity [19]. As a diluent, this molecule allows the system to crosslink while the viscosity is still lowered. HDDA has no stereoregularity, as the functional groups are positioned on both extremities of the molecule, meaning a more robust structure can be created with the presence of the second functional group. The lack of a chiral center allows for closer packing of polymer chains, strengthening the intermolecular attractions. The downside to the tight packing is the potential to bring higher shrinkage [38,39]. 

4-Acryloylmorpholine (ACMO) (Rahn AG, Zürich, Switzerland) and N-Vinyl-2-pyrrolidone (NVP) (Basf, Ludwigshafen, Germany) were introduced to formulations as a stiff diluent, responsible for dropping viscosity and providing high Young’s modulus. However, high concentrations of monofunctional such as ACMO and NVP can inhibit the mechanical potential of a binder, as they can only partake in the elongation of a polymer chain but not crosslink, weakening the structure’s stiffness [40]. Therefore, when developing the various formulations (Table 2), the diluent ratio was kept within 30–60 wt.% as it was believed to give the highest mechanical strength and suitable binder viscosity [19]. 

Then, 1.5 wt.% Bapo (Rahn AG, Zürich, Switzerland) photoinitiator was used per the manufacturer’s recommendation. The reactive diluents and photoinitiator were placed in a Clifton SW6H sonicator (Nickel-Electro, Weston-super-Mare, UK) for 20 min to ensure the effective dissolution of the photoinitiator [41]. The mixture was then mixed in a DAC 150FVZ-K speed mixer (Hauschild, Hamm, Germany) for 2 min at 1930 RPM. The crosslinkers were then added to the mixture and underwent high-speed mixing for 4 min at RPM of 1930 before being returned to the sonicator for 20 min to remove air bubbles [1].

A total of 7 formulations were created (Table 2), varying the concentration and presence of components to produce a low-viscous formulation suitable for high particle loading while maintaining high mechanical strength and stiffness. Each component’s contribution to these design goals was compared to find the ideal combination. 

A small sample of each binder was deposited onto a clear OHP film of 101 μm then exposed to UV light on an I-box Mono (Qidi Tech, Dongguan, China) with a wavelength of 405 nm. The excess uncured binder was wiped off and cleaned with Isopropyl Alcohol (IPA), and the layer thickness was measured with a micrometer. This process was repeated at different exposure times until a cure depth of double (x < +30–40 μm) the desired layer thickness (100 μm) was achieved. 

### 2.2. Printing Procedure

**Figure 3:**
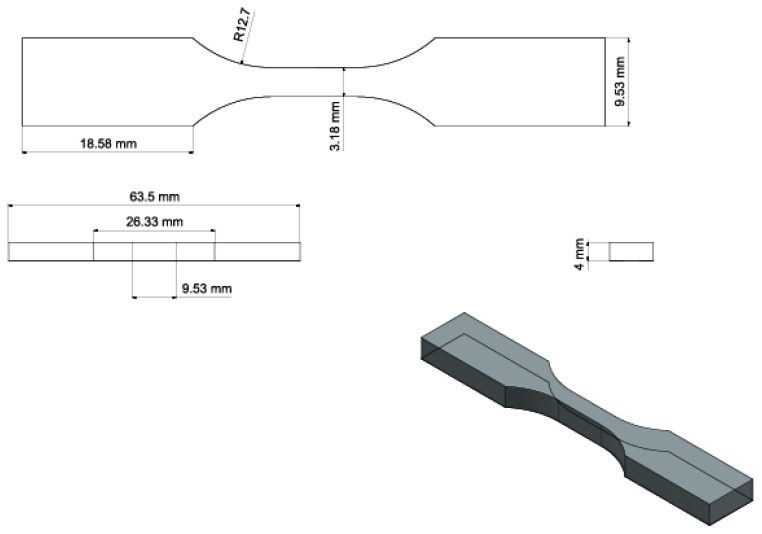
Drafting of ASTM D638 Type V tensile test bars.

The binders were loaded into a vat, and ASTM D638 [42], type 5 test samples (Figure 3), were printed at 100 µm print layer thickness, employing the appropriate exposure time. Bars were printed vertically to assess the weakest expected result for each formulation after post-curing [43]. After printing, the tensile bars were detached from the build platform and cleaned in a vat of IPA. 

### 2.3. Post-Curing Study 

**Figure 4:**
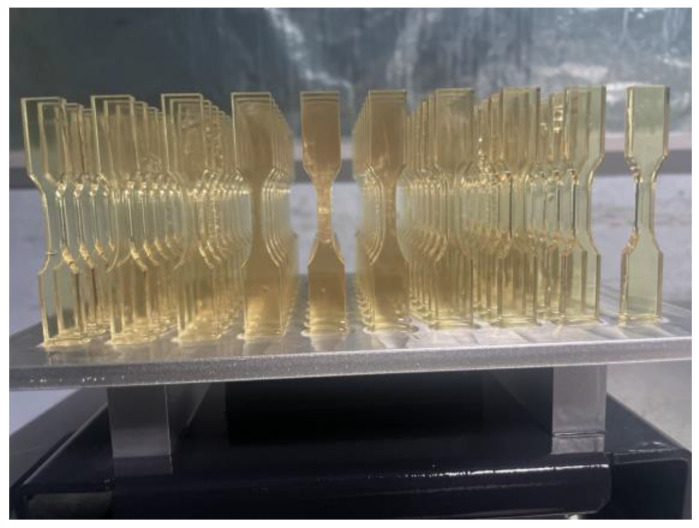
Printed type 5 tensile bars (ASTM-D638).

A post-cure study was conducted on the three best-performing formulations to study exposure conditions’ impact on their performance. Type 5 tensile bars were printed vertically at 50 μm layer thickness at the same exposure time used in the binder study (Figure 4) [44,45]. All formulations were post-cured at elevated temperatures and various periods using a Photocentric Cure L2, 405 nm UV light source convection and UV oven (Photocentric Ltd., Peterborough, UK) to study how each formulation’s mechanical properties varied with post-curing (Table 3).

### 2.4. Slurry Preparation 

Due to the rapid heating of heterogeneous systems during planetary mixing, the weighed Fe-6.5 wt%Si powder (x_d_ ≤ 45 μm) particle diameter and total loading of 43 *v*/*v*% and 64 *v*/*v*% was added incrementally to the binder and mixed in 30 s intervals to ensure sufficient mixing without excessive heating. 

### 2.5. Characterization Methods 

#### 2.5.1. Cure Depth 

Disks of a precise layer thickness (100 μm) were printed on the ibox Mono at 10 mm diameter starting at 10 ss, increasing to 60 s at 10 s intervals to compare curing behavior. Cured samples were cleaned with IPA before the thickness was measured using a micrometer. 

#### 2.5.2. Viscosity

The viscosity of formulations was measured using a Brookfield RST-CPS-P rheometer (Ametek, Braunstone Town, UK). Tests were carried out at a constant temperature of 25 °C, a shear rate of 800 s^−1^ for 120 s, creating 60 data points. Then, 0.2 ml of binder was deposited on the sampling surface for reading. For binders, an average of 60 data points was taken for fluid viscosity due to the Newtonian fluid behavior exhibited by homogenous systems. However, for slurries containing Fe-6.5 wt%Si particles suspended in the binder, the viscosity was taken at 100 s^−1^ due to potential non-Newtonian behavior causing variance in viscosity with applied shear.

#### 2.5.3. Tensile Testing

A Multitest- dV(u) (Mechmesin, West Sussex, UK) with 2.5 kN capacity was used to perform tensile tests at 1 mm/min. For each post-curing setting, including green bodies, seven bars were tested to gain an average for Ultimate Tensile Strength (UTS), MPa, Young’s Modulus (E), and strain at break, %. Stress at 5% strain, MPa, was also added as a mechanical property to study the binder’s predicted tensile strength at the point failure predicted for composite Fe-6.5 wt%Si flexural bars. 

#### 2.5.4. Hardness Testing and Surface Roughness 

Specimens were printed in the geometry set by ASME B46.1 standard [46] for hardness tested on a Shore D durometer (Sauter AG, Basel-Stadt, Switzerland). An average was taken for the center point of seven samples. The samples tested were of both green body and post-cured states to observe the effect of post-curing conditions on the hardness of the polymer. 

**Figure 5:**
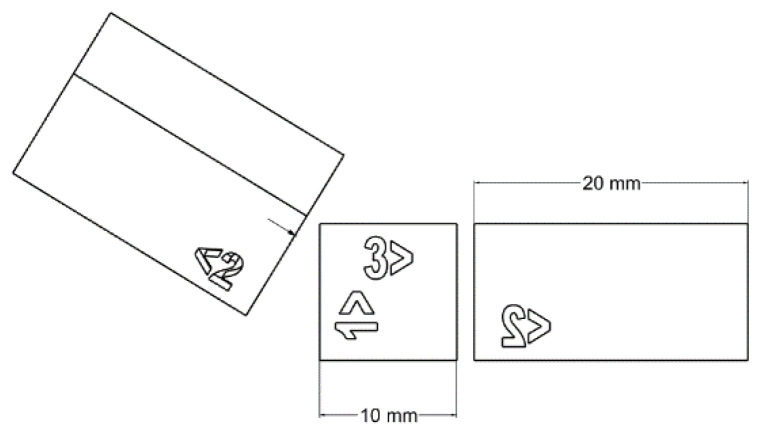
Drafting of hardness/surface roughness bars.

The same specimen geometry was used to measure the surface roughness of the green bodies and post-cured ones following ISO 4287 [47]. An area surface roughness was determined along the Z axis (printing direction). Figure 5 shows the dimensions and geometry of the bars. A numbering of 1 mm indentation was added to the bar to ensure surface roughness and hardness measurements were taken on the same face. Testing was conducted on a Bruker Alicona Infinite Focus (Bruker, Billerica, MA, USA). 

#### 2.5.5. FTIR-ATR

FTIR-ATR was used to study the degree of conversion and define the characteristics of formulations. Absorbance peaks were studied, and the area of peaks was used to determine the conversion of alkene bonds to an alkane, a sign of polymerization. To determine the degree of conversion, the area of C=C (alkene stretching) peaks at 1635 cm^−1^ were measured before and after exposure to UV. The area of the C=O vibration at 1720 cm^−1^ peak was taken as the baseline, as the peak is well-defined and uninfluenced by the photopolymerization in this system. The conversion was determined using Equation (1) [48]. Cured disks of 10 mm diameter and 100 µm thickness were created using a standard 5 s exposure time across the three top-performing formulations to test each binder’s reactivity comparatively.

(1) $$\xi =1-\left[\frac{{\left(\frac{A\left(C=C\right)}{A\left(C=O\right)}\right)}_{P}}{{\left(\frac{A\left(C=C\right)}{A\left(C=O\right)}\right)}_{U}}\right]$$

(*ξ*) Conversion; (*A*) Area of peak; (*P*) Polymerized sample; (*U*) Uncured sample. 

A Bruker Alpha Platinum-ATR (Bruker, MA, USA) was used to carry out FTIR-ATR on samples.

For uncured resin, a small amount was deposited directly on the measuring zone of the spectrometer, and for cured samples, the same disk geometry was used as the cure depth study. Before each test, the measurement surface was cleaned with IPA. A background measurement was taken to omit any noise produced by the surrounding atmosphere. FTIR-ATR was also used to estimate the functionality of each formulation by determining the peak area of uncured samples at 810 cm^−1^ to determine the abundance of alkene bonds available for conversion. This value was obtained through the peak area analysis of the 810 cm^−1^ absorption band for each binder at the uncured state and defined as the Maximum Saturation Potential (MSP). 

#### 2.5.6. Vibrating Sample Magnetometry (VSM)

To prepare the magnetic composite test samples for the VSM tests, the magnetic slurry was deposited on OHP film and exposed to UV light to achieve a cure depth of 20 μm. The excess material was wiped off, and a new layer of the uncured slurry was deposited over the previously cured layer. This process was repeated to achieve an accumulated thickness of 100 μm. Then, 2 mg of the magnetic composite was placed into a gelatin capsule, and the remaining space was filled with cotton wool to prevent the composite from moving. The sample was placed into the vibrator of a Cryogen-free measurement system (Cryogenic, London, UK) run on a helium cooling system with a magnetizing field of up to 2 T.

## 3. Results and Discussion

### 3.1. Binder Study

#### 3.1.1. Comparison of the Tensile Strength and Young’s Modulus of Formulations Containing ACMO or DPHA

Formulations F1 and F2 comprised the same components, except F1, which contained 10 wt.% of DPHA, while F2 held 10 wt.% ACMO. Both components were added to the formulation system to increase the Young Modulus of the material to achieve higher stress at lower strain. DPHA increases the crosslinking density due to increased functional groups available for polymerization [26,49]. ACMO is a monofunctional cyclic ether, commonly referred to as a stiff monomer, due to the partial dipole moment occurring as the electrons within the ether attract toward the oxygen in the molecule. Its presence in a binder formulation increased Young’s Modulus by creating strong intermolecular forces while the functional group partook in polymerization, creating strong covalent bonds with other components. Both components were compared to observe their performance in increasing the UTS and stiffness in as-print and post-cured conditions and their impact on the viscosity of the binders. 

Replacing the DPHA for ACMO led to a 50% decrease in viscosity (193 mPa·s, 96.5 mPa·s, respectively). However, F1 showed greater mechanical strength and stiffness at the green state than F2 (Table 4). This outcome was expected as covalent bonds resulting from DPHA are stronger than the hydrogen bonds formed with ACMO [50]. The increased abundance of functional groups from the presence of DPHA led to a higher degree of polymerization at the green state. Further crosslinking during the post-curing continued to increase the rigidity of the structure, leading to the parts made of F2 becoming increasingly brittle. Post-curing at 60 °C + UV for 30 min made specimens too brittle to produce tensile test results.

#### 3.1.2. Assessment of the Need for NVP in Formulations Already Containing ACMO

NVP is a monofunctional diluent introduced to formulations due to its low viscosity and high thermal stability. NVP is also known to extend conversion during co-polymerization systems [51], which is useful for VPP of highly reflective particles loaded resins as it more readily copolymerizes. However, NVP is known to accelerate degradation due to its rapid double-bond conversion rate [52]. 

Due to the similarities of NVP and ACMO in purpose, functionality, and viscosity (Table 1), the possibility of formulation simplification was explored. The 15 wt.% previously held by NVP in F2 was distributed between ACMO and HDDA. Then, 5 wt.% was added to HDDA to maintain low viscosity, while the remaining 10 wt.% was added to ACMO to enhance stiffness and tensile strength. This change was made to simplify the formulation, limit the increase in viscosity, and observe the impact the changes had on tensile strength and stiffness. 

The changes to create F3 led to a 53.4% increase in viscosity (96.5 mPa·s → 148 mPa·s). However, a higher mechanical performance was observed at the green state, and after the post-curing test. However, in the absence of NVP, mechanical properties were decreased when post-curing temperature was increased from 40 °C to 60 °C. The reduction in mechanical properties may be due to increased thermal motion compared to an NVP-containing formulation that suppresses thermal thinning behavior [53]. Ultimate tensile strength decreased at 60 °C post-curing with increased ACMO content, and the stiffness increased. Increased stiffness with decreased UTS and strain at break suggests that at 20 wt.%, ACMO brought brittleness. HDDA also supported crosslinking due to its difunctional nature, which increased Young’s modulus. 

#### 3.1.3. Lowering Binder Viscosity while Maximizing Tensile Strength 

Until this stage, the ratio of reactive diluent to primary crosslinker remained the same, as the focus was on finding suitable supporting components. However, as the primary component was also the component of the highest viscosity, formulations were explored where the content of the primary crosslinker was reduced to drop the overall binder viscosity while maximizing the mechanical strength. Although ACMO was favored over DPHA in Section 3.1.1, formulations were explored where both ACMO and DPHA were present to compensate for the lowered primary content with a high functionality component. 

To lower the viscosity and to make the binder more suited for high particle loading, the content of the primary crosslinker was reduced by 5 wt.%, and DPHA was reintroduced to maintain tensile strength through the abundance of functional groups, creating formulation F4. The benefits of this were reflected in tensile properties. DPHA prevented the decrease in performance seen with F3 by furthering the crosslinking process, giving higher UTS and Young’s Modulus at 60 °C+ UV post-curing. At this stage, it was concluded that ACMO and DPHA work harmoniously to improve the binder’s tensile properties; therefore, further investigation was needed to determine the ideal ratio of ACMO to DPHA (formulations F4, F5, and F6). 

### 3.2. Reactivity 

FTIR-ATR was used to define the abundance of potential sites for polymerization. The alkene twisting out of plane vibration, identifiable at 810 cm^−1^, exists due to adjacent functional groups that make up an acrylate [54], as the carbonyl group causes an electron withdrawal effect. Therefore, this peak alone can be used to identify alkene bonds on the monomers’ functional group. The significance of this value is that it assigns a quantitative value to the density of alkene bonds available for polymerization in formulations of many components of various concentrations and functionality. With this, an MSP value can be assigned to formulations. The MSP value for each formulation tested can be found in Table 5.

**Figure 6:**
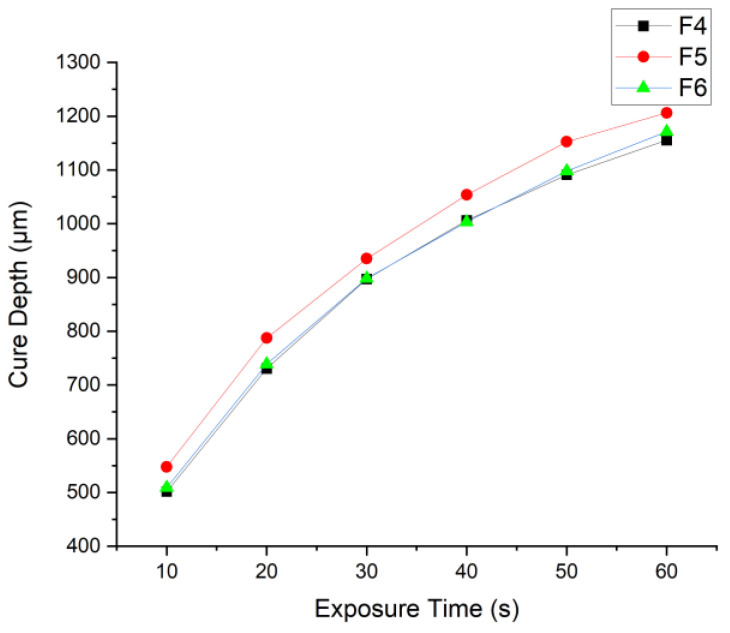
Cure depth results test of F4, F5, and F6.

Figure 6 shows the curing behavior of formulations F4, F5, and F6. All three formulations followed the same trend due to no particle loading and being exposed to the same light intensity with the same abundance of photoinitiator. F5 was found to have the highest performance in terms of cure depth across all time intervals above 10 s. While at 5 s of exposure, FTIR-ATR showed that the formulation with the greatest MSP also showed the highest degree of conversion. This result’s significance shows that the binder reactivity driving force varies with the progression of time.

The MSP was the significant binder characteristic at the starting point of polymerization. The system was stationary when irradiated with UV; therefore, the polymerization rate was sped up in the first few seconds of exposure by the abundance of C=C per unit area. However, when the system became energized by UV exposure, the molecules increased kinetic energy, increasing their rate of motion. At this stage, the viscosity became a more prominent factor in the polymerization rate. The distance that a radical needed to travel to find a region of unsaturation increased as polymerization progresses because there were fewer C=C bonds per unit area. Therefore, the higher viscosity of the binder acted as an inhibitor to the reaction rate as more forces acted against the flow of reactive species. This phenomenon was shown in the results, as F5 had a viscosity 47.59% lower than F4 and F6. However, in formulations F4 and F6, the viscosity was the same, reflected in the results by how closely related the cure depth results were; while the slight increase in performance seen in F6 in comparison to F4 can be linked to the MSP.

### 3.3. Post-Cure Study 

**Figure 7:**
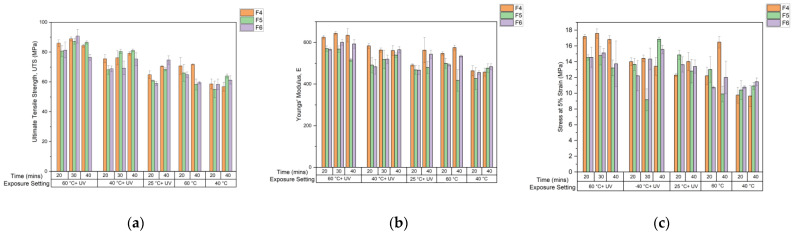
Post-cure study tensile test results. (**a**) Ultimate Tensile Strength, (**b**) Youngs’ Modulus, (**c**) Stress at 5% strain.

Following the post-cure study (Figure 7) conducted on F4, F5, and F6, the ideal post-curing setting to maximize the tensile properties of each formulation was 60 °C + UV for 30 min. It was found that 60 °C was a sufficiently high temperature to improve the mobility of uncured resin within the structure during post curing, reflected in the more viscous formulations, F4 and F6, when thermal post curing at 60 °C occurred. As the mobility of molecules increased further in F5 than in F4 and F6, the distance between interacting elements increased, elongating intermolecular bonds [55] and weakening the attraction, contributing to the falling of intermolecular forces. Therefore, in F5, the benefits of increased mobility were observed at a lower temperature of 40 °C. 

The greatest post-curing properties were observed when UV light was present as the polymerization reaction was UV initiated. Additional crosslinks can be formed during post-curing in the presence of UV light due to residual photoinitiator and uncured binder existing in the structure [56], providing greater mechanical strength to the tensile bars [57], as more energy is needed to break covalent bonds in comparison to weaker attractions such as van der Waals forces. However, in thermal post-curing at temperatures of <60 °C, energy supplied to the system was insufficient to initiate polymerization. When 60 °C + UV post-curing was applied, the best mechanical properties were achieved with 30 min of exposure [44,57]. Sufficient mobility was provided at 60 °C and the limit to crosslink formation and intermolecular forces appeared to be reached. Beyond this point, mechanical properties declined, suggesting a breakdown of intermolecular forces and bonds [58]. The increased crosslinking density of F6 appeared to have brought brittleness to the structure (Figure 7), as results showed a more drastic decline in UTS from 60 °C + UV for 30–40 min. 

At 25 °C + UV, the system’s dependency on MSP was reflected most. As 25 °C is not a sufficiently high temperature to increase molecule mobility, the system relied upon the presence of UV for crosslinking to improve mechanical properties. Regarding UTS, F6 was found to have the greatest increase in performance from 20 min to 40 min of exposure due to formulation’s high MSP. Large increases with exposure time were also observed in Young’s Modulus for F6. However, F4, the formulation with this higher proportion of primary crosslinker, gave the greatest stiffness value. 

### 3.4. Surface Roughness and Hardness

**Figure 8:**
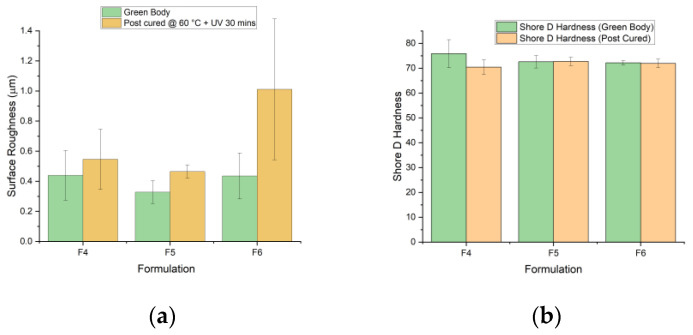
(**a**) Surface roughness results comparing green body and post-cured (60 °C + UV 30 min) samples across F4, F5, and F6. (**b**) Shore D Hardness test results of green and post-cured bodies (60 °C + UV 30 min).

Figure 8a shows the results obtained from the z-direction surface roughness analysis. An increase in surface roughness was observed in all three formulations between the green state and post curing. The warpage experienced with post curing was expected due to the continuation of crosslinking in the presence of UV, causing shrinkage to the part. Additionally, post curing at 60 °C caused thermal dilation [45], contributing to the increased surface roughness between the green body and post-cured parts. F5 showed the lowest levels of surface roughness at both green state and post-cured, as this formulation had the lowest (MSP). However, in F6, there was a great increase in MSP due to the abundance of DPHA content. The higher density of alkene bonds in the system during post curing would mean more bonds can form crosslinks during post curing, furthering the variations in dimensional accuracy. This phenomenon can be seen in F6, the formulation with the highest increase in surface roughness between the green and post-cured states. 

The results of the hardness test showed post curing not to have a significant impact on the hardness of formulation specimens. However, in F4, a 7% decrease in hardness was observed (75.83–70.43). In F5 and F6, shore hardness between green bodies and post-cured species was negligible. F4 contained the lowest quantity of stiff monomer, ACMO, and showed the greatest hardness decrease. F5 held the greatest amount of ACMO (30 wt%) and was the only formulation to show a net increase in hardness with post curing.

### 3.5. Loading Study 

Based on the data gathered, F5 and F6 were closest to the design requirements for particle loading. F6 showed high tensile properties and conversion due to its MSP, creating high crosslink density, while F5 showed superior performance in terms of viscosity with competitive tensile properties. Post curing the binder at 60 °C + UV for 30 min did not greatly impact the hardness but enhanced the strength of the material further. While both formulations shared similar mechanical performances, F5 progressed primarily due to its much lower viscosity and was used as the binder for particle loading. 

**Figure 9:**
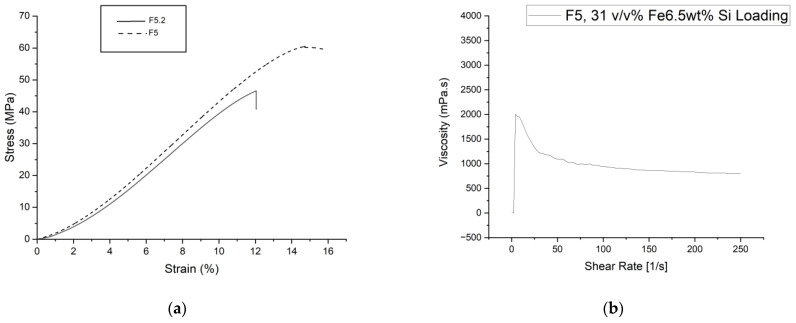
(**a**) Average green state tensile test results for type v dog bones produced from F5 and F5.1. (**b**) Rheology, viscosity data for 31 *v*/*v*% 20–45 µm diameter–F5 slurry.

The loading study used Fe-6.5 wt%Si FeSi particles ranging from 20 to 45 µm in diameter. A maximum of 31 *v*/*v*% particle loading was achieved using formulation F5 as the binder, as, beyond this point, the slurry’s viscosity exceeded 2 Pa.s. Additional loading to meet the design goal of >60 *v*/*v*% would make the viscosity too high for printing. Therefore, adjustments were made to the binder formulation to lower the viscosity further and allow loading to reach the target, which was 60 *v*/*v*%. Based on the findings of this study, the final formulation was made by making adaptations to the F5 formulation. Formulation F5.1 was produced by splitting the crosslinker proportions into (20 wt.% to the primary crosslinker and 25 wt.% to the secondary crosslinker). The ACMO abundance was also increased to 35 wt%. These changes provided a high MSP and very low viscosity formulation. A highly reactive formulation, capable of high Fe-6.5 wt%Si loading and stiff mechanical properties, could be achieved (Figure 9). The MSP was increased from 1.11 to 1.68 to increase the abundance of reactive species in polymerization, while the conversion with 5 s of exposure increased from 35.9% to 53.98%. As there was a 55.6% decrease in the content of the primary crosslinker, there was an anticipated drop in ultimate tensile strength and elongation at break (Figure 9). However, UTS and strain at break were deemed compromisable for comparable stress at 5% strain and increased particle-loading potential.

**Figure 10:**
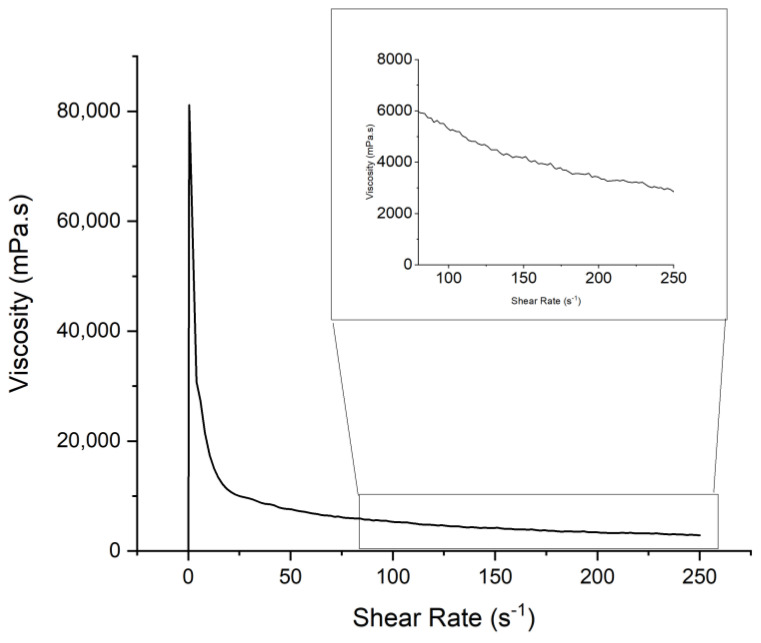
Viscosity data of F5.1 loaded with 64 *v*/*v*% Fe-6.5 wt%Si at <20 µm particle diameter.

The maximum loading achieved using formulation 5.1 was 64 *v*/*v*% (Figure 10), meeting the loading requirement of this study. Additional particle loading was expected to increase the viscosity of the slurry through the loading of smaller particle size (<20 µm), benefiting loading in terms of reaching shear thinning behavior at a lower shear rate in comparison to larger particles [59]. Shear thinning behavior is beneficial to the type of LCD masking printer intended for use with this slurry, wiper-assisted VPP, as the application of shear force on the slurry from the wiper reduces the viscosity making a new layer readily replenishable. However, as seen in the study conducted by Gaudio et al., with loading with a smaller particle size, the viscosity of the slurry at rest is much higher compared to systems with the same volumetric load but a larger particle size. 

### 3.6. Vibrating Sample Magnetometry (VSM)

**Figure 11:**
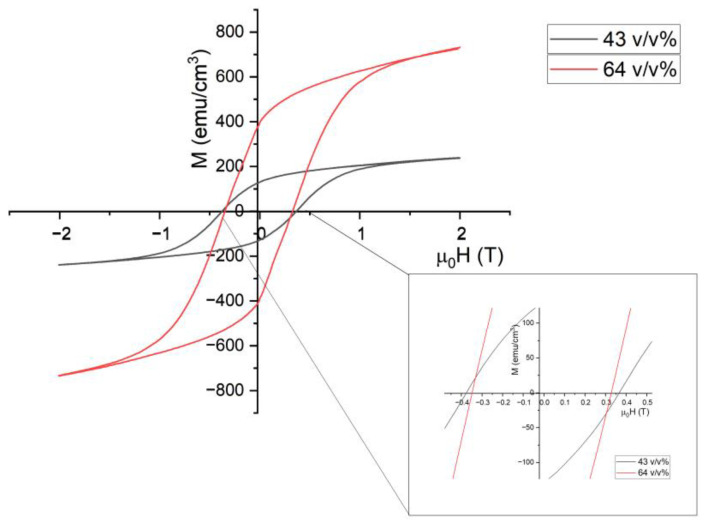
Magnetic hysteresis loop for Fe-6.5 wt%Si soft magnetic composite at 64 *v*/*v*% and 43 *v*/*v* % measured by VSM at 295 K.

Figure 11 shows magnetic hysteresis loops measured by the VSM at the temperature of 293 K. The results correspond to ferromagnetic behavior at room temperature. A significant difference in magnetization loops can be seen between the two volumetric loads of Fe-6.5 wt%Si. 

At a field of 2 T, the saturation magnetization observed in the 64 *v*/*v*% (Ferrosilicon in resin) composite was 733.43 emucm^−3^, whereas at 43 *v*/*v*%, it was 239.08 emucm^−3^. The percolation of the magnetic phase was applied to this system [60,61,62,63] to understand the difference between the two composites at different loads. In this case, the critical percolation threshold should be achieved at 60 *v*/*v*%, meaning the sample with 43 *v*/*v*% formed separate magnetic phase clusters, explaining much lower saturation magnetization. In contrast, the composite at 64 *v*/*v*% exceeded the predicted percolation threshold, leading to the 206.77% increase in saturation magnetization. This result suggests that the magnetic particles in the 64 *v*/*v*% composite were close enough to form interacting percolating clusters [64,65], while at 64 *v*/*v*%, there were still voids and gaps between the magnetic particles, which likely caused a reduction in the average exchange and dipolar interactions and resulted in lower saturation magnetization values compared to bulk Fe-6.5 wt%Si (1353 emu/cm^3^) [66,67]. 

The coercivity of the composites was determined through interpolation, giving a value of 0.348 T for the 64 *v*/*v*% composite and 0.376 T for the 43 *v*/*v*% composite. This 7% coercivity difference shows that the polymer did not cause a significant impact on the coercivity between the two loads. However, compared to the coercivity of bulk Fe-6.5 wt%Si (0.0012 T) [67], encapsulating the magnetic phase within the polymer matrix creates additional local magnetic defects and residual stress, usually leading to increased coercivity [67,68,69].

Table 6 shows the magnetic polarization of the 64 *v*/*v*% composite compared to soft magnetic composites from other research [67] and industry [70]. The data were collected to assess the suitability of VPP for the fabrication of magnetic composites for different applications [71]. In a study conducted by Wu et al. [67], Fe-6.5 wt%Si/SiO_2_ composites were fabricated using Fluidized Chemical Vapor Deposition (FCVD) and conventional in situ Chemical Vapor Deposition (CVD), which were then compared to bulk Fe-6.5 wt%Si. These three fabrication methods were compared to commercially available soft magnetic composite powder produced by Höganäs (Somaloy, Höganäs, Sweden).

All composites presented in Table 6 carried lower magnetic polarization as bulk Fe-6.5 wt%Si. The magnetic composites studied were not magnetizing enough for induction motor applications; however, all were suitable for power electronics. This result was expected due to the non-magnetic components causing insulation, weakening the magnetic properties. The magnetic composite produced using VPP held the lowest maximum saturation magnetization compared to other synthesis methods, expected as the method as composite was measured in its green state. The VPP composite retained the insulating polymer material, benefiting characteristics such as weather and chemical resistance, complex geometry, rapid and reduced energy intensity manufacturing, machinability, and less stiff material properties.

### 3.7. Future Studies

Following the findings of this study, the next stage of investigation is to improve the quality of the slurry by studying methodologies for slowing settlement rates and reducing the presence of agglomerates in the slurry. A study of dispersants and particle morphology will be conducted, comparing the improvement to rheology and printability to the impact changes to the slurry have on the magnetic properties.

## 4. Conclusions

This study investigated the formulation of a high-strength and low-viscosity binder for loading Fe-6.5 wt%Si to a minimum volumetric load of 60 *v*/*v*% to print composites using VPP. Formulations of various proportions of reactive diluents and secondary crosslinkers were created, and their conversion, rheological, and mechanical properties were studied. A post-curing study was conducted to find the most suitable parameters and study the effect of exposure time and energy on the mechanical properties of binder formulations. The magnetic properties of loaded composites were then compared. The following conclusions were made:

1. The primary crosslinker had the greatest impact on Young’s Modulus, while DPHA aided the ultimate tensile strength and mechanical performance with post-curing.
2. The driving force for polymerization varies with exposure time. However, at <10 s of exposure, the abundance of functional groups played a larger role in polymerization, while the binder viscosity took precedence above 10 s.
3. A significant difference in saturation magnetization between loading below and above the predicted percolation threshold can be observed, with coercivity decreasing with particle volumetric loading. The 64 *v*/*v*% composite produced with VPP showed 0.9 T of magnetization, which exceeds the maximum magnetization needed for power electronic applications (minimum of 0.5 T), showing promise for VPP as a manufacturing method for soft magnetic composites.

## Figures and Tables

**Table 1:** Characteristics of used oligomers and monomers, data provided by material manufacturers.

Component	Category	Functionality	Viscosity (mPa·s)	Density (gcm^−3^)	Refractive Index	Molecular Weight
PRIMARY	Crosslinker	2	10000	1.11	1.4843	-
DPHA	Crosslinker	6	7000	1.198	1.489	578
HDDA	Dilutant	2	10	1.01	1.465	226
NVP	Dilutant	1	2.4	1.043	1.48	111.1
ACMO	Dilutant	1	12	1.1185	1.5121	141.17

**Table 2:** Binder study identifiers and formulations.

ID	Primary (wt.%)	NVP (wt.%)	HDDA (wt.%)	ACMO (wt.%)	DPHA (wt.%)
F1	60	15	15		10
F2	60	15	15	10	
F3	60		20	20	
F4	55		20	20	5
F5	45		20	30	5
F5.1	20		20	35	25
F6	50		15	25	10

**Table 3:** Various parameters for post-cure study.

		1	2	3	4	5	6	7	8	9	10	11	12
	UV	✔	✔	✔	✔	✔	✔	✔	✔				
Temperature (°C)	25	✔	✔										
	40			✔	✔	✔				✔	✔		
	60						✔	✔	✔			✔	✔
Time (min)	20	✔		✔			✔			✔		✔	
	30				✔			✔					
	40		✔			✔			✔		✔		✔

**Table 4:** Tensile results of green body and post-cured binder type v tensile bars.

	Viscosity (mPa·s)	UTS (MPa)	Standard Deviation (%)	E	Standard Deviation (%)	σ_5%_ (Mpa)	Standard Deviation (%)	Strain at Break (%)	Standard Deviation (%)
**Green Body**									
**F1**	193	30.19	0.77	267.6	7.48	6.18	1.08	21.05	1.08
**F2**	96.5	27.72	2.58	253.48	33.18	7.43	2.83	22.95	1.03
**F3**	148	44.34	1.16	393.24	27.10	10.35	3.19	17.05	1.51
**F4**	145	60.99	2.17	545.81	18.92	15.36	1.34	16.32	1.05
**F5**	76	60.742	3.64	536.649	12.5	17.38	0.35	15.67	0.08
**F5.1**	32	50.1	3.29	508.69	20.8	15.56	0.34	13.43	0.98
**F6**	145	61.56	2.51	558.65	33.98	15.64	1.91	17.01	1.06
**Post Cure 40 °C + UV 30 min**									
**F1**		53.85	1.16	469.73	10.54	7.81	5.53	19.16	1.70
**F2**		57.92	1.26	466.74	15.29	7.74	1.69	18.85	1.06
**F3**		61.4	0.6	541.23	12.71	12.88	8.00	15.89	1.61
**F4**		70.43	1.46	614.5	9.98	9.08	0.84	17.1	2.02
**F5**		68.95	4.92	583.26	30.41	14.9	4.41	16.64	0.89
**F5.1**		76.78	3.53	722.9	39.68	21.83	3.85	13.03	1.03
**F6**		73.89	3.38	635.05	24.98	17.52	2.35	15.34	1.63
**Post Cure 60 °C + UV 30 min**									
**F1**		-		-		-		-	
**F2**		70.2	0.04	508.52	10.60	13.62	1.23	18.66	1.69
**F3**		65.67	1.35	592.89	25.94	10.93	4.83	16.71	1.73
**F4**		79.98	0.08	672.79	1.79	19.45	0.32	14.96	0.11
**F5**		80.46	4.01	678.75	10.99	17.98	1.55	15.1	1.85
**F5.1**		71.8	2.33	746.11	7.76	20.3	1.32	12.33	0.51
**F6**		74.52	0.51	717.64	10.68	18.84	1.52	13.35	1.23

**Table 5:** Rheological and kinetic results of F4, F5, and F6.

Formulation	F4	F5	F6
Viscosity (mPa·s)	145	76	145
Maximum Saturation Potential (MSP)	1.22	1.11	1.23
Conversion (%)	45.12	35.90	45.71

**Table 6:** Comparison of the magnetic polarization of different soft magnetic composites and industry requirements.

		µ_0_M (T)	Source
Applications			
Induction Motors		2	[71]
Power Electronics		0.5	[71]
Synthesis method			
VPP	43 *v*/*v*%	0.3	
	64 *v*/*v*%	0.9	
Fluidized Chemical Vapor Deposition		1.6	[67]
Conventional In Situ Chemical Vapor Deposition		1.5	[67]
Materials			
Bulk Fe-6.5 wt%Si		1.7	[67]
Höganäs Somaloy		1.5	[70]

## Data Availability

The research data can made available upon request from the corresponding author.
